# Pro-inflammatory cytokines and their epistatic interactions in genetic susceptibility to schizophrenia

**DOI:** 10.1186/s12974-016-0569-8

**Published:** 2016-05-13

**Authors:** Lekshmy Srinivas, Neetha N. Vellichirammal, Ann Mary Alex, Chandrasekharan Nair, Indu V. Nair, Moinak Banerjee

**Affiliations:** Human Molecular Genetics Laboratory, Rajiv Gandhi Centre for Biotechnology, Trivandrum, Kerala 695 014 India; Nair’s Hospital, Ernakulam, Kerala India; Mental Health Centre, Trivandrum, Kerala India

**Keywords:** Cytokines, Schizophrenia, Pro-inflammatory, Genetics, Polymorphism, Epistasis

## Abstract

**Background:**

In schizophrenia, genetic background may provide a substrate for intrinsic maldevelopment of the brain through environmental influences, by recruiting neurotrophic factors and cytokines, to trigger the changes that lead to impaired neuronal functions. Cytokines being the key regulators of immune/inflammatory reactions are also known to influence the dopaminergic, noradrenergic, and serotonergic neurotransmission. Therefore, functional polymorphisms in cytokine genes may result in imbalances in the pro- and anti-inflammatory cytokine production.

**Methods:**

We screened polymorphisms in pro- and anti-inflammatory cytokine genes using a case-control association study in a South Indian population. The role of allele, genotype, haplotype, and diplotypes of these cytokine genes and their epistatic interactions were assessed in contributing to the risk of developing schizophrenia. Meta-analysis for the reported associations was also monitored for global significance.

**Results:**

The pro-inflammatory cytokine gene polymorphisms in *IL1A*rs1800587, *IL6*rs1800796, *TNFA*rs361525, and *IFNG*rs2069718 were associated with schizophrenia. The study also provides significant evidence for strong epistatic interactions among pro-inflammatory cytokine genes *IL6* and *IFNG* in the development of schizophrenia. In silico analysis suggested that associated risk variants were indicative of altered transcriptional activity with higher production of IL1α, IL-6, TNF-α, and IFN-ɤ cytokines. Meta-analysis indicated heterogeneity among study population while *IL1A*rs1800587 was found to be globally significant.

**Conclusions:**

It is important to identify the nature of inflammatory response that can be amplified by the environment, to influence either Th1 response or Th2 response. The associated functional variants in the study are involved with increased expression resulting in higher production of the pro-inflammatory cytokines IL-1α, IL-6, TNF-α, and IFN-γ. The interaction of immunological stressors with these high producer alleles of pro-inflammatory cytokines may suggest that even a lower threshold may be sufficient to induce a resultant chronic effect on the psycho-social and environmental stressors that may result in the development and pathogenesis of schizophrenia. Understanding environmental factors that influence the expression of these pro-inflammatory cytokine genes or their interaction can possibly help in dissecting the phenotypic variation and therapeutic response to antipsychotics in schizophrenia.

**Electronic supplementary material:**

The online version of this article (doi:10.1186/s12974-016-0569-8) contains supplementary material, which is available to authorized users.

## Background

Schizophrenia is a severe and debilitating mental illness, affecting about seven to eight individuals per 1000 of the general population [1]. It is characterized by three broad spectrum behavioral domains such as positive symptoms noted as delusions and hallucinations and negative symptoms noted as apathy, anhedonia, social withdrawal, and cognitive domain [2]. These behavioral domains tend to vary in their presentation based on ethnicity, culture, or environmental conditions which might impact treatment and its outcome [3]. These facts make schizophrenia a complex disorder which involves multiple genes with mild to moderate effect and non-genetic risk factors like environmental and psychological assaults that alter the brain’s chemistry [4]. Numerous theories have been proposed regarding the cause of schizophrenia, ranging from developmental or neurodegenerative processes or neurotransmitter abnormalities to infectious or autoimmune processes. A number of environmental factors have been implicated for increased risk to schizophrenia, such as maternal infections [5], obstetric complications [6], neonatal hypoxia, and brain injury [7]. Besides these, schizophrenia patients experience elevated morbidity from infectious and autoimmune diseases [8]. These environmental risk factors recruit cytokines to the brain to mediate inflammatory processes [9]. Thus, cytokines being the key regulator of immune/inflammatory reactions and brain development emerge as a common pathway for genetic and environmental components of schizophrenia.

Cytokines can act as signals between cells to regulate the immune response to injury and infections [10]. Besides providing communication between immune cells, they also play a role in signaling the brain to produce neurochemical, neuroendocrine, neuroimmune, and behavioral changes [10]. Cytokines can strongly influence the dopaminergic, noradrenergic, and serotonergic neurotransmission [11]. Pathogenic nature of neurotransmission can be mediated by alterations in the equilibrium status of Th1/Th2 response resulting in schizophrenia. Elevated levels of certain cytokines have been reported both in the serum and cerebrospinal fluid (CSF) of patients with schizophrenia [12]. However, it is known that an individual’s immunological response involving the cytokine systems tends to differ, depending on genetic background as well as on the type of immunogen [13]. Therefore, just monitoring the cytokines levels can be misleading without considering the role of underlying polymorphisms in cytokine genes. Genetic background provides a substrate for intrinsic maldevelopment of the brain through environmental influences, by recruiting neurotrophic factors and cytokines, to trigger the changes that lead to impaired neural functions. Therefore, the aim of the present study was to identify the role of pro-inflammatory and anti-inflammatory cytokine gene polymorphisms in schizophrenia in South Indian population and to search for epistatic interactions among cytokine genes that may contribute to schizophrenia risk. Immune response is heavily influenced by environmental, socio-cultural, and ethnic factors. In order to minimize the role of these modulating factors, the present study is restricted to Malayalam-speaking populations from Kerala in South India.

## Methods

### Subject selection

A case-control association study was carried out using 248 schizophrenia patients (98 males and 146 females) with mean age group of 33 +/−11 years and 244 controls (83 males and 161 females) with mean age group of 33 +/−7 years that belonged to ethnically matched Malayalam-speaking population of Kerala, South India. These samples were genetically [14] and epigenetically [15] stratified based on their genetic architecture and similar environmental background as has been reported earlier. Patients were diagnosed as per the Diagnostic and Statistical Manual of Mental Disorders, 4th Edition (DSM-IV) criteria for schizophrenia by two psychiatrists who were a part of the study. Patients with major affective disorders and schizo-affective disorder were excluded. Patients with narcotic drugs or alcohol abuse were also excluded. All the participants who consented were included in the study. The study was approved by the Institutional Ethics Committee for Biomedical Subjects, according to the Indian Council of Medical Research (ICMR) guidelines. Five to ten milliliters of peripheral blood was collected and used for DNA isolation.

### Genotyping and functional assessment

Genomic DNA was isolated from lymphocytes using standard organic extraction method. Twenty-three polymorphisms including 22 SNPs and 1 VNTR (variable number tandem repeat) polymorphism from ten cytokine genes (*IL1A*, *IL1B*, *IL1RN*, *IL3*, *IL4*, *IL6*, *IL10*, *IFNG*, *TNFA*, and *TGFB1*) were screened. Genes and SNPs were selected based on their functional significance and previous reports on association with any disease conditions. A list of cytokine candidate genes and their selected polymorphisms indicating the SNP rsID, chromosomal position, locations, and predicted functional effects are given in Table 1. Functional effect predictions of SNPs were done using FuncPred, Functional SNP Prediction tool (snpinfo.niehs.nih.gov/snpinfo/snpfunc.htm). Functional prediction of the deleterious effect if any of the associated SNPs with respect to the functional categories such as protein coding, splicing regulation, transcriptional regulation, and post-translation was assessed in silico using F-SNP program (compbio.cs.queensu.ca/F-SNP/), FastSNP (fastsnp.ibms.sinica. edu.tw), SNPNexus (snpnexus. org), HaploReg (broadinstitute.org/mammals/haploreg), and regulomeDB (regulome.stanford.edu). F-SNP extracts information from large number of resources such as PolyPhen, SIFT, SNPeffect, SNPs3D, LS-SNP, Ensembl, ESEfinder, RescueESE, ESRSearch, PESX, TFSearch, Consite, GoldenPath, KinasePhos, OGPET, and Sulfinator to generate a functional significance (FS) score (Additional file 1: Table S1).

**Table 1:** List of cytokine candidate genes and their selected polymorphisms

Gene	Locus	SNP	rs id	GMAF	Alleles	Genotyping method	Location	Predicted functional effect
*IL1A*	2q13	−889G>A	rs1800587	0.2786	G/A	TaqMan AD (C_9546481_20)^a^	Promoter	TFBS, splicing (ESE or ESS)
*IL1B*	2q13	+3954 C>T, Phe105Phe	rs1143634	0.1328	C/T	PCR-RFLP	Exon 4	sSNP, splicing (ESE or ESS)
		−31T>C	rs1143627	0.4724	C/T	PCR-RFLP	Promoter	TFBS
		−511C>T	rs16944	0.4906	T/C	PCR-RFLP	Promoter	TFBS
*IL1RN*	2q13	(86)_n_ VNTR	rs2234663		1/2	PCR FLP	Intron 2	Regulatory protein-binding sites
*IL4*	5q31.1	−590C>T	rs2243250	0.4698	C/T	PCR-RFLP	Promoter	TFBS
		−33C>T	rs2070874	0.4012	C/T	TaqMan AD (C_16176215_10)^a^	Promoter	TFBS
*IL6*	7p21	−597G>A	rs1800797	0.1382	G/A	PCR-RFLP	Promoter	TFBS
		−572G>C	rs1800796	0.3139	G/C	PCR-RFLP	Promoter	TFBS
		−174G>C	rs1800795	0.1412	G/C	PCR-RFLP	Promoter	TFBS
*IL10*	1q31	−592A>C	rs1800872	0.4349	T/G	PCR-RFLP	Promoter	TFBS
		−819C>T	rs1800871	0.4347	G/A	KASPar assay	Promoter	TFBS
		−1082G>A	rs1800896	0.2722	T/C	KASPar assay	Promoter	TFBS
*IL3*	5q31.1	rs31400T>C	rs31400	0.4581	T/C	TaqMan AD (C_3141153_10)^a^	Promoter	Promoter/regulatory region
		rs31480C>T	rs31480	0.2883	C/T	TaqMan AD (C_2397270_10)^a^	Promoter	TFBS
		rs40401C>T, Ser27Pro	rs40401	0.4195	C/T	TaqMan AD (C_2397269_30)^a^	Exon 1	nsSNP, benign
*TNFA*	6p21.3	−308G>A	rs1800629	0.0903	G/A	KASPar assay	Promoter	TFBS
		−238G>A	rs361525	0.0609	G/A	KASPar assay	Promoter	TFBS
*IFNG*	12q24.1	+3232A>G	rs2069718	0.3832	A/G	TaqMan AD (C_15799728_10)^a^	Intron 3	–
		+874A>T	rs2430561	0.2802	A/T	KASPar assay	Intron 1	NFKB binding site
*TGFB1*	19q13.2	+915G>C, Arg25Pro	rs1800471	0.0483	G/C	KASPar assay	Exon 1	nsSNP, benign
		+869A>G, Leu10Pro	rs1800470	0.4547	A/G	TaqMan AD (C_22272997_10)^a^	Exon 1	nsSNP
		−509C>T	rs1800469	0.368	C/T	KASPar assay	Promoter	TFBS

*TFBS* transcription factor binding site, *ESE* exonic splicing enhancer, *ESS* exonic splicing silencer, *sSNP* synonymous SNP, *nsSNP* nonsynonymous SNP, *GMAF* global minor allele frequency

^a^TaqMan allelic discrimination accession numbers

The genotyping method for each polymorphism is given in Table 1. Wherever PCR-RFLP and FLP was carried out, their PCR primers, PCR condition, restriction enzymes, and their allele sizes for each polymorphism are shown in Additional file 1: Table S2. Few SNPs were genotyped using TaqMan allelic discrimination assay (Applied Biosystems, Foster City, CA) according to conditions recommended by the manufacturer in an Applied Biosystems 7500 or Applied Biosystems 7900HT Real-Time PCR. The real-time-based KBiosciences (Hoddesdon, UK) Competitive Allele-Specific PCR genotyping system (KASPar) was also used according to manufacturer’s instructions for genotyping in an Applied Biosystems 7500 Real-Time PCR.

### Statistical analysis

Allelic, genotypic, haplotypic, and diplotypic frequencies were calculated and compared using Unphased v3.0.13 software. Association was also analyzed using dominant, recessive, and additive genetic models. Subgroup analysis based on gender was also carried out. Deviations from the Hardy-Weinberg equilibrium (HWE) were tested for all polymorphisms in controls by comparing observed and expected genotype frequencies using Haploview v4.1 program (www.broad.mit.edu/mpg/haploview). The linkage disequilibrium (LD) structure among SNPs and haplotype blocks was visualized by generating LD plots using the Haploview.

Gene-gene interactions among cytokine genes associated with schizophrenia were detected by the open-source multifactor dimensionality reduction (MDR) software package, version 3.0.1, available at http://www.epistasis.org/software.html. The Tuned ReliefF (TuRF) filter algorithm was used to remove noisy SNPs from the pool of possible candidates. Using this procedure, we analyzed the top 4 SNPs in order to understand the best two-way, three-way, or four-way gene-gene interactions. This reduces the chance of overfitting the data, which is possible when considering high-order interactions (e.g., >4) in relatively small datasets. The new one-dimensional multi-locus genotype variable was evaluated for its ability to classify and predict disease status using cross-validation and permutation testing. The model with maximum testing accuracy (TA) and cross-validation consistency (CVC) was selected as the best model. Statistical significance was evaluated using a 1000-fold permutation test using multifactor dimensionality reduction permutation testing module (MDRPT v1.0.2 beta) software. Finally, we used measures of interaction information to provide a statistical interpretation of the gene-gene interaction models. To visualize the nature and strength of interactions among polymorphisms, an interaction dendrogram was used. We initially used the whole dataset on cytokine genes for dendrogram construction. The presence of LD between SNPs on the same gene may affect the interaction analysis. Therefore, to reduce the effect when SNPs were in strong pair-wise LD, defined as *r*^2^ > 0.8, one of the pair was randomly dropped. This new multi-locus attribute now represented the combined effect of all SNPs in a gene and constructed a refined interaction dendrogram.

A meta-analysis with the random-effects and fixed-effects model was performed for previously studied SNPs using Review Manager 5.2 (reviewmanager.software. informer.com/5.2/). All the previous studies published in Pubmed indexed journals which explored similar variants in genes involved in pro- and anti-inflammatory cytokines were included in this study. Family- or sibling-based association studies, non-interpretable data and articles in non-English language were excluded from the study. The meta-analysis included the most studied SNPs in immune response, *IL1A*rs1800587 (G-889 A), *IL6* rs1800795 (G-174C), and *TNFA* rs1800629 (G-308 A) and rs361525 (–238G>A) (Additional file 1: Table S3). The inconsistency index *I*^2^ was used to assess between-study heterogeneity. A *p* value of <0.05 was considered as significant throughout the analyses.

## Results

**Fig. 1:**
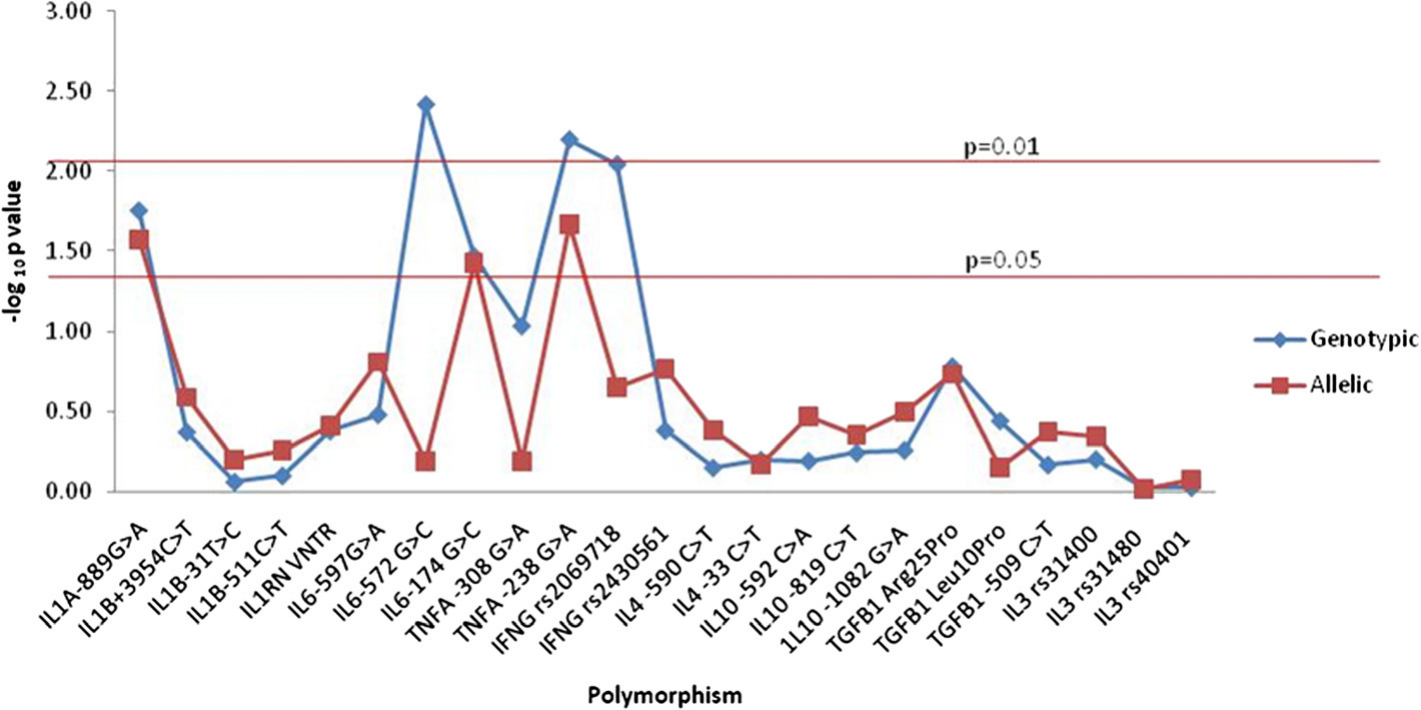
Overview of association of cytokine gene polymorphisms with schizophrenia. X-axis shows the polymorphisms screened, and Y-axis shows the *P* values for each polymorphism on a logarithmic scale

The association of polymorphisms in pro-inflammatory and anti-inflammatory cytokine genes with schizophrenia is presented in Fig. 1. Among cytokine genes, significant association was observed with pro-inflammatory cytokine gene polymorphisms of *IL1A*, *IL6*, *TNFA*, and *IFNG* with schizophrenia in our study population (Table 2). The *IL1A* -889 G>A polymorphism was found to be associated at both allelic and genotypic level with schizophrenia. The allele A of the *IL1A* -889 polymorphism was significantly higher in cases (*P* = 0.026; OR = 1.36; CI = 1.04–1.79). The *IL1A* -889 genotypes also showed an association with statistical significance (*P* = 0.04) with increased frequency of AA genotype. This genotypic association enhanced further in a recessive model (AA vs. GG+GA) with schizophrenia (*P* = 0.017, OR = 2.08, CI = 1.12–3.84). In silico prediction indicated altered transcriptional activity with functional significance score of 0.21. The allele and genotype frequencies of the SNPs in *IL1B* and VNTR in *IL1RN* did not differ significantly between the cases and the controls (Additional file 1: Table S4). The *IL1B* -31 and -511 loci in both patient and control groups were in near complete linkage disequilibrium (*r*^2^ = 0.98 in controls and *r*^2^ = 0.97 in patients).

**Table 2:** Genotype and allele frequencies of the associated pro-inflammatory cytokine genes and their functional significance score

Polymorphism	Genotype	Patients	Controls	*P* value	Model-based *P* value/OR (CI)	Allele	Patients	Controls	*P* value/OR (CI)	FS score
IL1A -889G>A	GG	102 (0.42)	118 (0.49)			G	313 (0.64)	344 (0.71)		
rs1800587	GA	109 (0.45)	108 (0.44)	0.04	0.017^a^	A	175 (0.36)	142 (0.29)	0.026	0.21
	AA	33 (0.14)	17 (0.07)		^a^2.1 (1.08–4)				1.36 (1.04–1.8)	
IL6 -597G>A	GG	165 (0.67)	149 (0.61)			G	402 (0.82)	382 (0.78)		
rs1800797	GA	72 (0.29)	84 (0.34)	0.38		A	90 (0.18)	106 (0.22)	0.18	
	AA	9 (0.04)	11 (0.05)							
IL6 -572G>C	GG	62 (0.25)	81 (0.33)			G	268 (0.54)	273 (0.56)		
rs1800796	GC	144 (0.59)	111 (0.45)	0.015	0.003^b^	C	224 (0.46)	215 (0.44)	0.64	
	CC	40 (0.16)	52 (0.21)		^b^1.7 (1.18–2.42)					
IL6 -174G>C	GG	177 (0.72)	153 (0.63)			G	415 (0.84)	385 (0.79)		
rs1800795	GC	61 (0.25)	79 (0.33)	0.10	0.034^c^	C	77 (0.16)	101 (0.21)	0.04	0.40
	CC	8 (0.03)	11 (0.05)		^c^1.5 (1.03–2.2)				1.4 (1.02–1.96)	
TNFA -308G>A	GG	202 (0.82)	192 (0.78)			G	443 (0.9)	435 (0.89)		
rs1800629	GA	39 (0.16)	51 (0.21)	0.09		A	49 (0.1)	53 (0.11)	0.64	
	AA	5 (0.02)	1 (0.01)							
TNFA -238G>A	GG	199 (0.80)	216 (0.89)			G	445 (0.9)	457 (0.94)		
rs361525	GA	47 (0.19)	25 (0.1)	0.019	0.006^d^	A	49 (0.1)	29 (0.06)	0.02	0.21
	AA	1 (0.01)	2 (0.01)		^d^2.05 (1.2–3.45)				1.74 (1.08–2.8)	
IFNG +3232A>G	AA	85 (0.35)	61 (0.25)			A	285 (0.58)	265 (0.55)		
rs2069718	AG	115 (0.47)	143 (0.59)	0.026	0.009^f^, ^f^1.6 (1.1–2.29)	G	203 (0.42)	221 (0.45)	0.22	ND
	GG	44 (0.18)	39 (0.16)		0.019^e^, ^e^1.6 (1.08–2.36)					
IFNG +874A>T	AA	96 (0.39)	107 (0.44)			A	300 (0.61)	316 (0.65)		
rs2430561	AT	108 (0.44)	102 (0.42)	0.40	0.40	T	192 (0.39)	168 (0.35)	0.16	
	TT	42 (0.17)	33 (0.14)							

*FS score* functional significance score

^a^Recessive model (AA vs. GG+GA); ^b^Additive model (GC vs. GG+CC); ^c^Recessive model (GG vs. GC+CC); ^d^Additive model (GA vs. GG+AA); ^e^Recessive model (AA vs. AG+GG); ^f^Additive model (AG vs. AA+GG)

In *IL6*, -572 G>C (rs1800796) was found to be associated with schizophrenia at the genotypic level (*P* = 0.015) with an increased frequency of heterozygous genotype. In an additive model (GC vs. GG+CC), this association enhanced further (*P* = 0.003, OR = 1.69, CI = 1.18–2.42). *IL6* -174G>C (rs1800795) G allele was found to be associated with schizophrenia (*P* = 0.037, OR = 1.41, CI = 1.02–1.96). The GG genotype was observed to be in higher frequency in patients but in a recessive model (GG vs. GC+CC), this association with schizophrenia turned significant (*P* = 0.034, OR = 1.51, CI = 1.03–2.21). Diplotype analysis showed that the combination of *IL6* -572 GC and the major genotype of *IL6* -174 GG genotypes were strongly associated (*P* = 0.001, OR = 1.5). In silico prediction indicated altered transcriptional activity for *IL6* -572 G>C (rs1800796) and *IL6* -174G>C (rs1800795) with functional significance score of 0.21 and 0.39, respectively.

In *TNFA*, -238G>A (rs361525) polymorphism was found to be significantly associated at the genotypic and allelic levels with schizophrenia. The allele A was significantly higher in cases when compared to controls (*P* = 0.021, OR = 1.74, CI = 1.08–2.82). The genotype distribution in patients and controls differed significantly (*P* = 0.019) with higher frequency of heterozygous genotype in patients. This association was observed significant (*P* = 0.01, OR = 1.93) in dominant model (AA+AG vs. GG) but enhanced further in an additive model (GA vs. GG+AA) (*P* = 0.0059 OR = 2.05, CI = 1.22–3.45). In silico prediction indicated altered transcriptional activity for this SNP with a functional significance score of 0.21. *TNFA* -308 G>A polymorphism was not found to be associated with schizophrenia at allele and genotype level. But at haplotypic level, the presence of *TNFA* -308 G allele along with *TNFA* -238 A allele was significantly associated (*P* = 0.02, OR = 1.75) with schizophrenia. Significant diplotypic association was also observed with the presence of -308 GG genotype along with -238 GA genotype (*P* = 0.01, OR = 2.05).

*IFNG* rs2069718 was found to be associated with schizophrenia in our population at the genotypic level with increased frequency of AA genotype in schizophrenia patients (*P* = 0.026). This association enhanced further in a recessive model (AA vs. AG+GG)-based association (*P* = 0.019, OR = 1.59, CI 1.08–2.36), while the heterozygous AG genotype had a significant protective effect (*P* = 0.009, OR = 1.60, CI 1.12–2.29). Using subgroup analysis based on gender, we observe a significantly strong association with *IFNG* rs2069718 (*P* < 0.001) with AA as risk genotype for females.

The anti-inflammatory cytokine genes *IL4*, *IL10* and *TGFB1*, and *IL3* were not found to be associated with schizophrenia in our South Indian Kerala Population. The observed allele and genotype frequencies for these cytokine gene SNPs are given in Additional file 1: Table S4. In silico prediction of transcriptional activity of the studied SNPs with their functional significance score is mentioned in Additional file 1: Table S1. Interestingly, the associated SNPs were found to have higher functional significance for altered transcriptional activity.

### Gene-gene interactions—MDR analysis

**Fig. 2:**
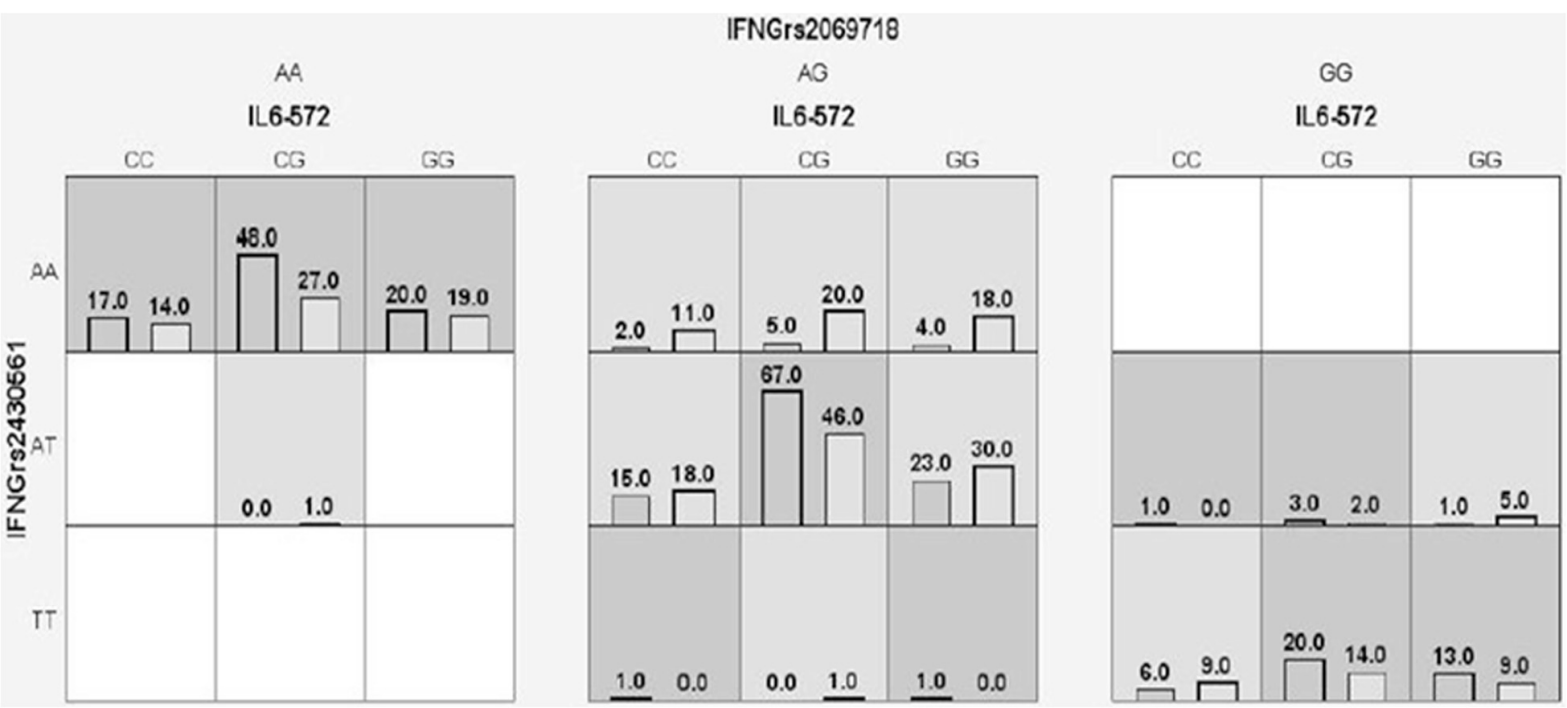
Distribution of cases and controls for the three-locus genotype combinations of *IL6* -572, *IFNG* rs2430561, and *IFNG* rs2069718 associated with high risk and low risk for schizophrenia using MDR analysis (*left bar* in cell indicates cases and *right bar* indicates controls)

**Fig. 3:**
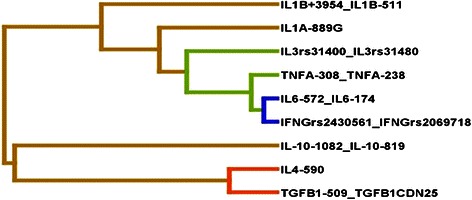
MDR interaction dendrogram showing interactions of pro-and anti-inflammatory cytokine genes

The four best SNPs selected by the TuRF filter algorithm were *IL6* -572G>C, *IL6* -174G>C, *IFNG* rs2430561, and *IFNG* rs2069718. An exhaustive MDR analysis that evaluated all possible combinations of these four polymorphisms was carried out. Table 3 summarizes the gene-gene interactions among cytokine gene polymorphisms predicted using MDR and shows the best model on each order. The *IL6* -572G>C turns out to be the best single-locus model, and it can be seen having significant epistatic interactions with *IFNG* SNPs (*P* = 0.0095) with testing accuracy of 0.5837, CVC of 10/10. Figure 2 summarizes the distribution of cases and controls for the three-locus genotype combinations of *IL6*-572, *IFNG* rs2430561, and *IFNG* rs2069718 associated with high and low risk for schizophrenia. The pattern of high-risk and low-risk cells differs across each of the multi-locus dimensions. Such differences are evidence of a gene-gene interaction. The three SNPs in the three-factor model, which formed the overall best model in our analysis, i.e., *IL6* -572, *IFNG* rs2430561, and *IFNG* rs2069718, were combined using the attribute construction function to create a new single multi-locus attribute (*IL6-572_IFNGrs2430561_ IFNGrs2069718*) to the dataset. Next, we did a forced analysis with that single constructed attribute and the statistics for the analysis of that variable was obtained. Interestingly, this new single multi-locus attribute had a high testing accuracy of 0.6182 along with 10/10 cross-validation consistency and a very significant permutation *P* value <0.001. This can further be visualized by tree diagram indicating that the pro-inflammatory cytokine genes were clustered on the same branch and their proximity in the tree diagram clearly indicates that these genes may interact together to modulate the risk of schizophrenia (Fig. 3).

**Table 3:** Gene-gene interactions predicted using MDR

Best combination in each order	TA	CVC	*P* value
*IL6*-572	0.562	10/10	0.100
*IFNG*rs2430561, *IFNG*rs2069718	0.5783	10/10	0.018
*IL6*-572, *IFNG*rs2430561, *IFNG*rs2069718^a^	0.5837	10/10	0.0095
*IL6*-572, *IL6*-174, *IFNG*rs2430561, *IFNG*rs2069718	0.5739	10/10	0.0305

*TA* testing accuracy, *CVC* cross-validation consistency

^a^Best model

### Meta-analysis

**Fig. 4:**
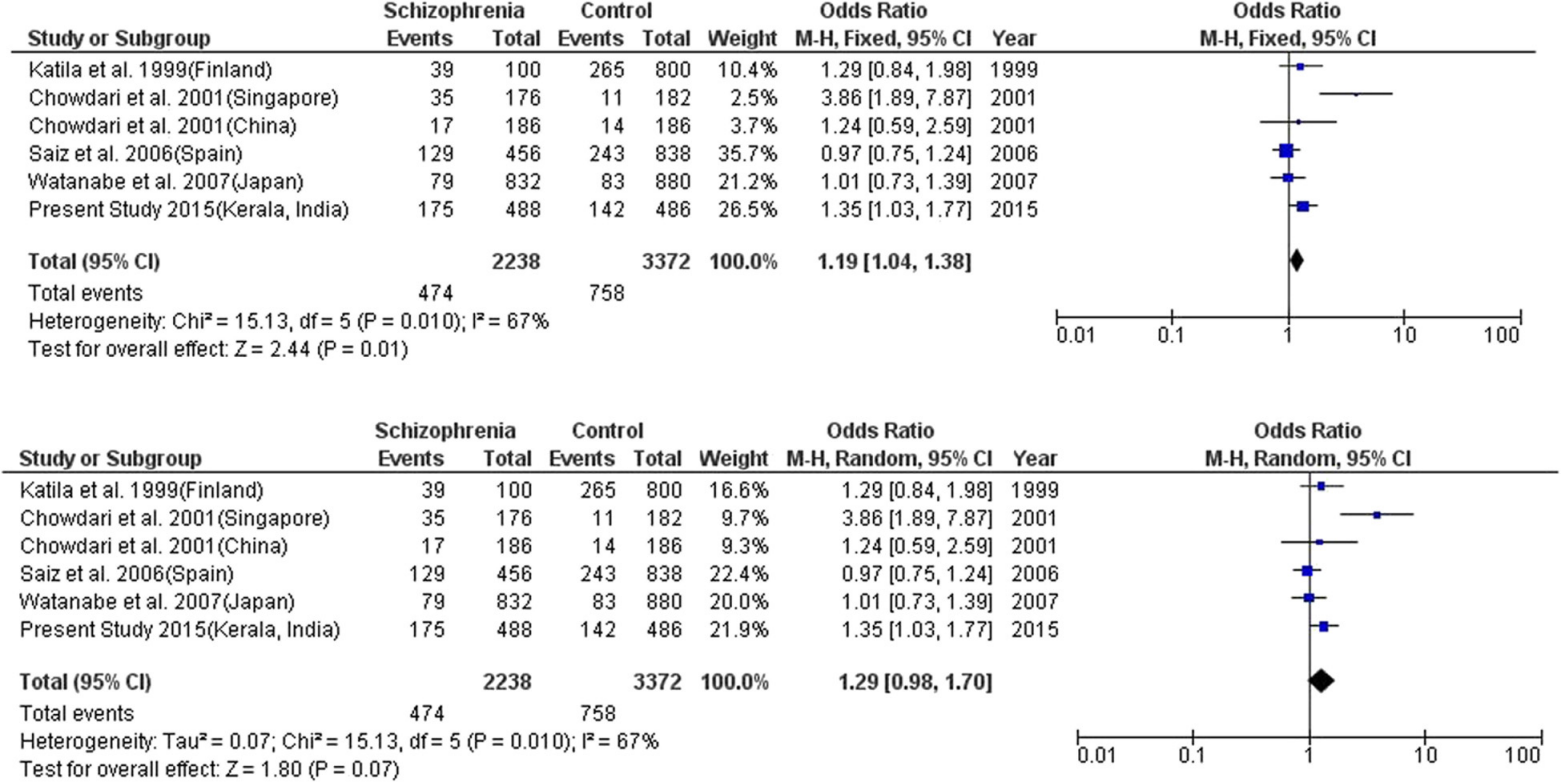
Meta-analysis of *IL1A* rs1800587 risk allele A versus G allele in schizophrenia patients in comparison to normal control using fixed and random model

We performed a meta-analysis for all the SNPs in the present study with the previous reports for the same SNPs in different ethnicities. For *IL1A* rs1800587, an overall significant association (*P* = 0.01) was observed in the fixed-effects model (Fig. 4). However, higher heterogeneity (67 %) was observed between the studies. To reduce the heterogeneity, a possible method is to restrict the analysis to the studies done in the Asian populations. The overall effect significantly increased (*P* = 0.003), but the heterogeneity remains unaltered. Similarly, we performed meta-analysis with *IL6* rs1800795 (-174G>C) while no sufficient data was available for *IL6* rs1800796 (-572G>C). Overall effect was found to be negative with no association but was indicative of high heterogeneity among the study population (*I*^2^ = 77 %) (Additional file 2: Figure S1). Majority of data on IL6 polymorphisms come from European population. Interestingly, *IL6* rs1800795 (-174G>C) and rs1800796 (-572G>C) were strongly associated in our south Indian population alone. Meta-analysis with *TNFA* rs1800629 (-308 G>A) and rs361525 (-238G>A) was carried out with none of the SNPs showing any significant overall effect (Additional file 3: Figure S2, Additional file 4: Figure S3). Both these SNPs indicated high heterogeneity among study population. Extensive studies have been carried out with *TNFA* rs1800629 (-308 G>A) while studies with rs361525 (-238G>A) is just emerging. Our positive association with rs361525 (-238G>A) might indicate significance of this SNP to be replicated in larger ethnic population.

## Discussion

In the present study, we report significant association of SNPs in pro-inflammatory cytokine genes *IL1A*, *IL6*, *TNFA*, and *IFNG* with schizophrenia in the Malayalam-speaking Dravidian population of India. Contrary to findings from other populations, we did not observe association of polymorphisms in anti-inflammatory cytokine genes with schizophrenia, in our study population. Equilibrium between pro- and anti-inflammatory cytokine is essential to maintain the homeostasis in the system. Pro-inflammatory cytokines such as IL-1α, IL-6, TNF-α, and IFN- γ are responsible for early responses and amplify inflammatory reactions, whereas anti-inflammatory cytokines, which include IL-4, IL-10, and TGF-β, have the opposite effect, in that they limit the inflammatory responses. The association of pro-inflammatory cytokine genes with schizophrenia in our population validates the hypothesis of excess of pro-inflammatory cytokine in the pathophysiology of schizophrenia. This could relate to the elevated levels of pro-inflammatory cytokines in patients with schizophrenia.

IL-1α is a pro-inflammatory cytokine, and we observe a strong association with *IL1A* -889 SNP. The *IL1A*-889 SNP is in the promoter region which has a predicted transcription factor binding site. We found an association of *IL1A* -889 A allele and AA genotype with schizophrenia in our population. *IL1A* -889 allele A is reported to be associated with a fourfold increase of IL-1α production [16], and the GG genotype is associated with lower transcriptional activity and lower levels of circulating IL-1α, in comparison to AA genotype [17]. *IL1* has been described as an astroglial growth factor that has a role in brain development [18]. It can also modify the metabolism of neurotransmitters or influence neural development [19]. Thus, the association of *IL1A* high producer genotype AA and allele A with schizophrenia may imply alterations in the early developmental process of brain or neural development or modify the metabolism of neurotransmitters. In meta-analysis, *IL1A* rs1800587 was found to be significantly associated from global perspective (*P* = 0.01), which enhanced further when the association was evaluated from the Asian population perspective (*P* = 0.003). However, higher heterogeneity among the study population was observed. An association with the A allele was observed in two study population, i.e., Singapore and Kerala (present study), in India. Interestingly, both these places lie below the tropic of cancer near to the equator as compared to the rest of the study populations that lie above the tropic of cancer. This might indicate how environment shapes our cytokine gene selection process. A comparison of the allelic frequencies of the SNP among the various world populations also suggests a similar pattern.

With IL-6, a multifunctional cytokine with pro-inflammatory, immunoregulatory, and neuroprotective activities, we report a strong association of *IL6*-174 G allele and GG genotype and *IL6*-572 GC genotype with schizophrenia in South Indian population. The change from a G to C at position 174 creates a potential binding site for the transcription factor neurofibromatosis type I (NF-1), which comprises a family of structurally related transcription factors active in many cell types [20]. The G allele of *IL6*-174 SNP was reported to be associated with higher expression levels [20]. Both individuals homozygous or heterozygous for the G allele have been shown to have higher IL-6 levels in the plasma, higher *IL6* gene transcriptional activity, and higher inducible IL-6 responses, when compared to subjects homozygous for the C allele [20, 21]. In our population, we found an association of the high producer allele G and GG genotype of this SNP with schizophrenia. Thus, overexpression of pro-inflammatory cytokine *IL-6* may be a factor for schizophrenia development in our population. A study in the Polish population suggests that the *IL6* -174G>C is associated with the risk of paranoid schizophrenia [22], while a Chinese study revealed no association of the SNP with schizophrenia [23]. In Armenian population, an association of rs1800795 (-174G/C) with schizophrenia and corresponding blood level of IL-6 in patients was found to be 1.5-fold higher than in controls [24]. Meta-analysis with *IL6* rs1800795 (-174G>C) was indicative of having no effect from global perspective and was also suggestive of high heterogeneity among study populations. Interestingly, we report association with the high producer G allele which is in contrast to the associated allele in European population. Meta-analysis could not be carried out for *IL6* rs1800796 (-572G>C) due to lack of sufficient data. However, genome-wide linkage analysis has identified the chromosome 7p21.1–22.3 region, which contains the *IL6* gene, as one of the susceptibility loci to schizophrenia [25]. IL-6 is not only synthesized and released in immune cells of the peripheral blood; it is also produced by activated astrocytes and microglia cells in the central nervous system (CNS). In the CNS, IL-6 has been found to regulate brain development, synaptic plasticity, and various behaviors related to feeding, sleep, and stress [26]. A strong relationship between IL-6 and neurotransmitter production has been reported by various studies. IL-6 can stimulate neurons in vitro to secrete dopamine [27]. The peripheral application of IL-6 in animal experiments enhanced the dopaminergic and serotonergic turnover in the hippocampus and frontal cortex, without affecting noradrenaline [28]. These observations point towards direct influence of IL-6, on the catecholaminergic neurotransmitter system which might be mediated by environmental effects on the high producer allele.

Tumor necrosis factor-alpha (TNF-α) is a pro-inflammatory cytokine, which is secreted in the CNS by neurons, glial cells, and astrocytes [29]. We found a significant association of *TNFA* -238G>A (rs361525) polymorphism with higher prevalence of allele A and heterozygous genotype GA with schizophrenia. *TNFA* -238G>A located in the promoter region has a potential transcription binding site. Earlier studies with *TNFA* polymorphisms have reported contradictory observation with schizophrenia in various ethnic populations, while few studies have reported positive associations [30–32] and equal number of studies have failed to replicate these positive associations of *TNFA* polymorphisms in schizophrenia [33–35]. These discrepancies indicate the role of ethnic variation as majority of the positive associations were reported in Caucasian populations while lack of association was observed predominantly in populations of East Asian origin. Meta-analysis with *TNFA* rs1800629 (-308 G>A) and rs361525 (-238G>A) indicate lack of any significant impact of these SNPs to have a global effect. Extensive heterogeneity among study population might indicate ethnicity-specific impact in influencing their role in the disease. Studies with rs361525 (-238G>A) is just emerging and our positive association with this SNP might indicate significance of this SNP to be replicated in larger ethnic population. The positive association in the present study could be of significance as TNF-α has been demonstrated to have cytotoxic effects on embryonic mesencephalic dopaminergic neurons [36], which are suggested to have an important role in the etiopathogenesis of schizophrenia. The various effects of TNF-α suggest that disturbances in its levels may affect the functioning of the brain. TNF-α has been shown to have a stimulatory effect on the catecholaminergic system, whereas chronic TNF-α release induces an inhibitory effect [37]. This is in line with the theory that acute schizophrenia involves a hyperdopaminergic state with positive psychotic symptoms, whereas chronic schizophrenia with predominantly negative symptoms is associated with a relative hypodopaminergic state [38]. *TNFA* gene is located within the major histocompatibility complex (MHC) region at the short arm of chromosome 6 (6p21.3), which is implicated in linkage [39] and genome-wide association studies (GWAS) studies [40–42] in genetic susceptibility to schizophrenia. Thus, the *TNFA* gene can be considered a positional as well as a functional candidate gene in the search for genetic factors contributing to the development of schizophrenia.

IFN-γ is a pro-inflammatory cytokine, which is important for regulating immune and inflammatory events. IFN-γ is known to upregulate both class I and II MHC antigen expression on a variety of cells. IFN-γ can enhance the function of microglia by increasing the production of some cytokines, e.g., TNF-α, nitric oxide, and of free radicals [43], and thus play an important role in the induction of various central nervous system pathologies. Therefore, association of *IFNG* rs2069718 AA genotype with schizophrenia might be of significance. Earlier reports have shown a gender-specific association of *IFNG* +874A>T (rs2430561) polymorphism with paranoid schizophrenia in males, but not in females [44]. Reduced IFN-γ production has been reported to be associated with acute exacerbation of schizophrenia [45]. Elevated levels of IFN-γ were reported to be associated with positive symptoms of paranoid schizophrenia while the negative symptoms were associated with downregulated IFN-γ [46]. IFN-γ also inhibits serotonin release [47]. These evidences strongly indicate that *IFNG* rs2069718 may play a role in immune changes occurring in schizophrenia.

Collectively, these genetic associations with multiple pro-inflammatory cytokines, by interacting with environmental insults, may result in slow and progressive damage to brain, precipitating in schizophrenia. In our gene-gene interaction analysis, *IL6* and *IFNG* were found to have strong epistatic interaction. The proximity of clustering of pro-inflammatory cytokine genes indicates that these genes interact together to modulate the risk of schizophrenia. In fact, this interaction between the pro-inflammatory cytokine genes may reflect the production of cytokines at molecular and cellular level too. IL-6 is secreted by a wide range of cells including fibroblasts, monocytes, B cells, endothelial cells, T cells, microglia, and astrocytes. Depending upon the cell type, synthesis of IL6 is known to be induced by a variety of agents including other pro-inflammatory cytokines IL-1, TNF-α, and IFN-γ [48]. Observations on genetic association in the present study also indicate that pro-inflammatory cytokines are more likely to be amplified through molecular heterotic effect of *IL6* and *TNFA* towards the risk of developing schizophrenia in South Indian population. Molecular heterosis is becoming increasingly evident in complex disorders [49] which can impact at both transcriptional and translational level [50]. Therefore, it is important to identify the nature of inflammatory response that is amplified by the environment, and accordingly, may indicate whether the shift will be towards Th1 response or Th2 response. Complementary lines of evidence imply that dysregulation of pro-inflammatory cytokines plays a key role in schizophrenia through various psycho-social and environmental stressors. Prenatal exposure to a number of adverse factors—such as maternal infection and diabetes—is known to result in elevated levels of maternal pro-inflammatory cytokines which can mediate adverse effects on fetal brain development [51]. The pro-inflammatory mediators favor the production of quinolinic acid and are also known to impair the ability of astrocytes to remove glutamate [52]. Therefore, glutamate and quinolinic acid through their excitatory activity may induce neurotoxic effects through NMDA receptor agonism. Interestingly, the antipsychotics are also known to inhibit the release of pro-inflammatory cytokines [53]. Therefore, one would presume that the therapeutic response in schizophrenia in South Indian patients may be contributed through immune regulatory pathway. Cytokine expression and modulation are heavily influenced by environmental factors. The present study indicates that the high-producer alleles of pro-inflammatory cytokines are key to the development of schizophrenia in South India. Interaction of environmental stressors with these high-producer alleles of pro-inflammatory cytokines may suggest that even a lower threshold of these stressors may be sufficient to induce an effect that may result in schizophrenia. However, none of the associated SNPs remain significant after correction for multiple testing; therefore, they should be considered suggestive of their role in schizophrenia. The limitation of the study is with large effect size, and this would involve increased sample size. Large effect sizes are uncommon in complex diseases; therefore, multiple testing in schizophrenia and all the more for immune response genes may not hold promise. Increasing sample size would mean sampling genetically non-stratified sample or phenotypically divergent samples which will further compromise the observation. This is also the reason why genetic studies are not replicable across ethnicities, and this conflict is also evident in the meta-analysis which indicates high heterogeneity.

## Conclusions

It is important to identify the nature of inflammatory response amplified by the environment, whether the shift will be towards Th1 response or Th2 response. The present study provides evidence of strong independent associations of pro-inflammatory cytokine genes *IL1A*, *IL6*, *TNFA*, and *IFNG* with schizophrenia and their interactions in Dravidian South Indian population. The associated functional variants in the study are involved with increased expression resulting in higher production of the pro-inflammatory cytokines IL-1α, IL-6, TNF-α, and IFN-γ. The interaction of immunological stressors with these high-producer alleles of pro-inflammatory cytokines may suggest that even a lower threshold may be sufficient to induce a resultant chronic effect on psycho-social and environmental stressors that may result in the development of schizophrenia. These pro-inflammatory mediators are likely to induce excitatory activity through the production of quinolinic acid and impair the ability to remove glutamate resulting in neurotoxic effects. These findings support the hypothesis that genetically determined changes in cytokine regulation may contribute to the pathogenesis of schizophrenia. Understanding environmental factors that influence the expression of these pro-inflammatory cytokine genes or their interaction can possibly help us in dissecting the resultant phenotypic variation in schizophrenia. Interestingly, the antipsychotics are also known to inhibit the release of pro-inflammatory cytokines. Therefore, one would presume that the therapeutic response in schizophrenia in South Indian patients may be contributed through immune regulatory pathway.

## Ethics approval and consent to participate

The study was approved by the Institutional Ethics Committee for Biomedical Subjects, according to the Indian Council of Medical Research (ICMR) guidelines.

## Consent for publication

The manuscript does not contain any individual data.

## Availability of data and materials

The data will be shared on request based on the guidelines of Institutional Ethics Committee.

## Additional files

Additional file 1: Table S1. In silico functional prediction of relevant SNPs in this study using F-SNP program. **Table S2.** PCR conditions and polymorphism detection through RFLP for cytokine gene polymorphism. **Table S3.** List of studies included in meta-analysis. **Table S4.** Genotype and allele frequencies of polymorphisms that show lack of association with schizophrenia. (DOCX 41 kb) Additional file 2: Figure S1. Meta-analysis of *IL6* rs1800795 risk allele G versus C allele in schizophrenia patients in comparison to Normal control using fixed and random model. (TIF 347 kb) Additional file 3: Figure S2. Meta-analysis of *TNFA* rs361525 risk allele A versus G allele in schizophrenia patients in comparison to Normal control using fixed and random model. (TIF 354 kb) Additional file 4: Figure S3. Meta-analysis of *TNFA* rs1800629 A versus G allele in schizophrenia patients in comparison to Normal control using fixed and random model. (TIF 272 kb)

## Abbreviations

CNS: central nervous system
CSF: cerebrospinal fluid
CVC: cross-validation consistency
DSM-IV: Diagnostic Statistical Manual of Mental Disorders, 4th Ed
GWAS: genome-wide association studies
HWE: Hardy-Weinberg equilibrium
ICMR: Indian Council of Medical Research
KASPar: competitive allele‐specific PCR system
LD: linkage disequilibrium
MDR: multifactor dimensionality reduction
MDRPT: multifactor dimensionality reduction permutation testing module
MHC: major histocompatibility complex
TA: testing accuracy
TuRF: Tuned ReliefF
VNTR: variable number tandem repeat
