# Supplementary material for: Pro-inflammatory cytokines and their epistatic interactions in genetic susceptibility to schizophrenia
Source: J Neuroinflammation. 2016 May 13;13:105. doi: 10.1186/s12974-016-0569-8 (PMC4866417; doi:10.1186/s12974-016-0569-8)
Supplement: Additional file 1: Table S1. — In silico functional prediction of relevant SNPs in this study using F-SNP program. Table S2. PCR conditions and polymorphism detection through RFLP for cytokine gene polymorphism. Table S3. List of studies included in meta-analysis. Table S4. Genotype and allele frequencies of polymorphisms that show lack of association with schizophrenia. (DOCX 41 kb) [file 12974_2016_569_MOESM1_ESM.docx]

**Supplementary Table 1.** In-Silico functional prediction of relevant SNPs in this study using F-SNP program

|  | Functional Category | Prediction Tool | Prediction Result | FS  score |
| --- | --- | --- | --- | --- |
| TNFArs361525 | transcriptional_regulation | TFSearch | changed | 0.208 |
|  |  | Consite | changed |  |
|  |  | GoldenPath | not exist |  |
| TNFArs1800629 | transcriptional_regulation | TFSearch | changed | 0.208 |
|  |  | Consite | changed |  |
|  |  | GoldenPath | not exist |  |
| IL3rs31480 | transcriptional_regulation | TFSearch | changed | 0.242 |
|  |  | GoldenPath | exist |  |
| TGFB1rs1800469 | transcriptional_regulation | TFSearch | changed | 0.208 |
|  |  | Consite | changed |  |
|  |  | GoldenPath | not exist |  |
| IL6rs1800795 | transcriptional_regulation | Ensembl-NS | frameshift_coding | 0.398 |
|  |  | GoldenPath | not exist |  |
|  |  | Ensembl-TR | regulatory_region |  |
| IL6rs1800796 | transcriptional_regulation | TFSearch | changed | 0.208 |
|  |  | Consite | changed |  |
|  |  | GoldenPath | not exist |  |
| IL1Ars1800587 | transcriptional_regulation | TFSearch | changed | 0.208 |
|  |  | GoldenPath | not exist |  |
| IL1Brs1143627 | transcriptional_regulation | Ensembl-NS | frameshift_coding | 0.5 |
|  |  | TFSearch | changed |  |
|  |  | GoldenPath | exist |  |
|  |  | Ensembl-TR | regulatory region |  |
| IL1Brs16944 | transcriptional_regulation | TFSearch | not changed | 0.065 |
|  |  | GoldenPath | exist |  |
| IL10rs1800896 | transcriptional_regulation | GoldenPath | exist | 0.101 |
| IL10rs1800871 | transcriptional_regulation | GoldenPath | exist | 0.101 |

FS Score; Functional significance score

F-SNP:<http://compbio.cs.queensu.ca/F-SNP/>

**Supplementary Table 2. PCR conditions and polymorphism detection through RFLP for cytokine gene polymorphism**

| **Gene / polymorphism** | **Primer Sequences** | **Annealing Temperature** | **RE** | **Fragments (bp)** |
| --- | --- | --- | --- | --- |
| ***IL1B*+3954C>T** | 5’-GTTGTCATCAGACTTTGACC-3’ | 52^0^C | *TaqI* | C:136+114 |
|  | 5’-TTCAGTTCATATGGACCAGA-3’ |  |  | T: 250 |
| ***IL1B*-31T>C** | 5’- TCTTTTCCCCTTTCCTTTAACT-3’ | 60^0^C^#^ , 50^0^C | *AluI* | C: 234 |
|  | 5’- GAGAGACTCCCTTAGCACCTAGT-3’ |  |  | T: 150 + 84 |
| ***IL1B* -511C>T** | 5’-TGGCATTGATCTGGTTCATC-3’ | 50^0^C | *AvaI* | C: 191+114 |
|  | 5’-GTTTAGGAATCTTCCCACTT-3’ |  |  | T: 305 |
| ***IL1RN* VNTR** | 5'-CTCAGCAACACTCCTAT-3' | 52^0^C | FLP | A1: 410 |
|  | 5'-TCCTGGTCTGCAGGTAA-3' |  |  | A2: 240 |
|  |  |  |  | A3: 500 |
|  |  |  |  | A4: 325 |
|  |  |  |  | A5: 595 |
| ***IL4* -590 C>T** | F: 5’-TAAACTTGGGAGAACATGGT-3' | 50.9^0^C | *AvaII* | C: 177+ 18 |
|  | R: 5’-TGGGGAAAGATAGAGTAATA-3' |  |  | T: 195 |
| ***IL6* -597G>A** | F: 5’-CTCCTCTAAGTGGGCTGAAG-3’ | 54^0^C | *FokI* | G: 212bp |
|  | R: 5’-CAAGCCTGGGATTATGAAGA-3’ |  |  | A: 125 + 87 |
| ***IL6* -572 C>G** | F: 5 ’-CTCCTCTAAGTGGGCTGAAG-3’ | 56.7^0^C | *BsrBI* | C: 212 |
|  | R: 5’-CAAGCCTGGGATTATGAAGA-3’ |  |  | G: 139+73 |
| ***IL6* -174 G>C** | F: 5 ’-TTGTCAAGACATGCCAAGTGCT-3’ | 59.1^0^C | *NlaIII* | G: 227 |
|  | R: 5’-GCCTCAGAGACATCTCCAGTCC-3’ |  |  | C: 118+109 |
| ***IL10* -592 C>A** | F: 5’-CTCAGTTAGCACTGGTGTAC-3’ | 55^0^C | *RsaI* | C: 480 |
|  | R: 5’-TGTTCCTAGGTCACAGTGAC-3’ |  |  | A: 240 |

^#^Touchdown PCR. Ramp at -1°C for each cycle for 10 cycles at 60^0^C. Add 25 cycles at 50^0^C.

RE, Restriction enzyme; FLP, Fragment Length Polymorphism detected using 3% agarose gel electrophoresis

**Supplementary Table 3:** List of studies included in meta-analysis

| **GENE** | **SNP** | **Author** | **Population** | **Sample size**  **Case/control** | **Year** |
| --- | --- | --- | --- | --- | --- |
| *IL1A* | rs1800587 (-889 G> A) | Katila et al | Finland | 100/800 | 1999 |
|  |  | Chowdari et al | China | 186/186 | 2001 |
|  |  | Chowdari et al | Singapore | 176/182 | 2001 |
|  |  | Saiz et al | Spain | 456/836 | 2006 |
|  |  | Watanabe et al | Japan | 832/880 | 2007 |
|  |  | Present Study | Kerala, India | 488/486 | 2015 |
| *IL6* | rs1800795 (–174G>C ) | Paul-Samojedny et al | Poland | 192/240 | 2010 |
|  |  | Zakharyan et al | Armenia | 206/210 | 2012 |
|  |  | Paul-Samojedny et al | Polish | 230/270 | 2013 |
|  |  | Debnath et al | Indian Bengalee | 200/200 | 2012 |
|  |  | Present Study | Kerala, India | 492/486 | 2015 |
| *TFNA* | rs361525  (–238G>A) | Shirts et al | USA | 488/552 | 2006 |
|  |  | Duan et al | China | 628/680 | 2004 |
|  |  | Pae et al | Korea | 304/304 | 2006 |
|  |  | Watanabe et al | Japan | 530/848 | 2007 |
|  |  | Debnath et al | Indian Bengalee | 200/200 | 2012 |
|  |  | Present Study | Kerala, India | 494/486 | 2015 |
|  | rs1800629  (–308G>A ) | Boin et al | Italy | 168/276 | 2001 |
|  |  | Riedel et al | Germany | 314/372 | 2002 |
|  |  | Meira-Lima et al | Brazil | 372/1314 | 2003 |
|  |  | Pae et al | Korea | 482/250 | 2003 |
|  |  | Tan et al | Singapore | 604/304 | 2003 |
|  |  | Tsai et al | China | 410/384 | 2003 |
|  |  | Duan et al | China | 628/680 | 2004 |
|  |  | Hashimoto et al | Japan | 594/916 | 2004 |
|  |  | Hanninen et al | Finland | 298/786 | 2005 |
|  |  | Kampman et al | Finland | 188/196 | 2005 |
|  |  | Shirts et al | USA | 488/552 | 2006 |
|  |  | Zai et al | Canada | 298/298 | 2006 |
|  |  | Pae et al | Korea | 304/304 | 2006 |
|  |  | Sacchetti et al | Italy | 646/692 | 2007 |
|  |  | Watanabe et al | Japan | 530/848 | 2007 |
|  |  | Czerski et al | Poland | 696/702 | 2008 |
|  |  | Betcheva et al | Bulgaria | 370/368 | 2009 |
|  |  | Naz et al | Pakistan | 200/140 | 2011 |
|  |  | Paul-Samojedny et al | Polish | 230/270 | 2013 |
|  |  | Present Study | Kerala, India | 492/488 | 2015 |

**Supplementary Table 4. Genotype and allele frequencies of polymorphisms that show lack of association with schizophrenia**

| **SNP Loci** | **Genotype** | **Patients** | **Controls** | **p** | **Allele** | **Patients** | **Controls** | **p** |
| --- | --- | --- | --- | --- | --- | --- | --- | --- |
| **IL1B +3954C>T** | **CC** | 173 (0.71) | 186 (0.76) |  | **C** | 411 (0.85) | 425 (0.87) | 0.258 |
|  | **CT** | 65 (0.27) | 53 (0.22) | 0.429 | **T** | 75 (0.15) | 63 (0.13) |  |
|  | **TT** | 5(0.02) | 5(0.02) |  |  |  |  |  |
| **IL1B -31T>C** | **CC** | 85 (0.35) | 90 (0.37) |  | **C** | 290(0.59) | 296 (0.61) | 0.637 |
|  | **CT** | 120 (0.49) | 116 (0.48) | 0.877 | **T** | 198 (0.41) | 190 (0.39) |  |
|  | **TT** | 39 (0.16) | 37 (0.15) |  |  |  |  |  |
| **IL1B -511C>T** | **TT** | 83 (0.34) | 90 (0.37) |  | **T** | 286 (0.59) | 295 (0.6) | 0.557 |
|  | **TC** | 120 (0.49) | 115 (0.47) | 0.802 | **C** | 202 (0.41) | 193 (0.4) |  |
|  | **CC** | 41 (0.17) | 39 (0.16) |  |  |  |  |  |
| **IL1RN VNTR** | **1/1** | 119 (0.5) | 133 (0.55) |  | **1** | 335 (0.7) | 350 (0.72) | 0.387 |
|  | **1/2** | 97(0.4) | 84 (0.35) | 0.422 | **2** | 145 (0.3) | 134 (0.28) |  |
|  | **2/2** | 24(0.1) | 25(0.1) |  |  |  |  |  |
| **IL4 -590 C>T** | **CC** | 167(0.68) | 159(0.70) |  | **C** | 406(0.83) | 381(0.84) | 0.464 |
|  | **CT** | 72(0.29) | 63(0.28) | 0.677 | **T** | 86(0.17) | 71(0.16) |  |
|  | **TT** | 7(0.03) | 4(0.02) |  |  |  |  |  |
| **IL4 -33 C>T** | **CC** | 167(0.68) | 172(0.71) |  | **C** | 407(0.83) | 410(0.84) | 0.586 |
|  | **CT** | 73(0.3) | 66(0.27) | 0.811 | **T** | 85(0.17) | 78(0.16) |  |
|  | **TT** | 6(0.02) | 6(0.02) |  |  |  |  |  |
| **IL10 -592 C>A** | **AA** | 71(0.29) | 62(0.25) |  | **A** | 259(0.52) | 240(0.49) | 0.340 |
|  | **AC** | 117(0.47) | 116(0.48) | 0.648 | **C** | 237(0.48) | 248(0.51) |  |
|  | **CC** | 60(0.24) | 66(0.27) |  |  |  |  |  |
| **IL10 -819 C>T** | **TT** | 73(0.3) | 62(0.26) |  | **T** | 257(0.53) | 242(0.5) | 0.444 |
|  | **TC** | 111(0.45) | 118(0.49) | 0.576 | **C** | 231(0.47) | 240(0.5) |  |
|  | **CC** | 60(0.25) | 61(0.25) |  |  |  |  |  |
| **IL10 -1082 G>A** | **AA** | 164(0.67) | 150(0.62) |  | **A** | 398(0.81) | 379(0.78) | 0.318 |
|  | **AG** | 70(0.28) | 79(0.32) | 0.556 | **G** | 96(0.19) | 107(0.22) |  |
|  | **GG** | 13(0.05) | 14(0.06) |  |  |  |  |  |
| **TGFB1 Arg25Pro** | **GG** | 209 (0.85) | 215 (0.88) |  | **G** | 454 (0.92) | 459 (0.94) | 0.185 |
|  | **GC** | 36 (0.14) | 29 (0.12) | 0.165 | **C** | 40 (0.08) | 29 (0.06) |  |
|  | **CC** | 2(0.01) | 0(0) |  |  |  |  |  |
| **TGFB1 Leu10Pro** | **AA** | 82 (0.34) | 71 (0.29) |  | **A** | 270 (0.55) | 263 (0.54) | 0.703 |
|  | **AG** | 106 (0.43) | 121 (0.5) | 0.365 | **G** | 218 (0.45) | 223 (0.46) |  |
|  | **GG** | 56 (0.23) | 51 (0.21) |  |  |  |  |  |
| **TGFB1 -509 C>T** | **CC** | 103 (0.42) | 92(0.38) |  | **C** | 304 (0.62) | 288 (0.6) | 0.416 |
|  | **CT** | 98(0.4) | 104(0.43) | 0.661 | **T** | 184 (0.38) | 194 (0.4) |  |
|  | **TT** | 43 (0.18) | 45(0.19) |  |  |  |  |  |
| **IL3 rs31400** | **TT** | 98(0.4) | 93 (0.38) |  | **T** | 312(0.63) | 297(0.61) | 0.457 |
|  | **TC** | 116 (0.47) | 111 (0.46) | 0.633 | **C** | 180(0.37) | 189(0.39) |  |
|  | **CC** | 32 (0.13) | 39 (0.16) |  |  |  |  |  |
| **IL3 rs31480** | **CC** | 124 (0.5) | 121 (0.49) |  | **C** | 349 (0.7) | 344 (0.7) | 0.964 |
|  | **CT** | 101 (0.41) | 102 (0.42) | 0.951 | **T** | 147 (0.3) | 144 (0.3) |  |
|  | **TT** | 23 (0.09) | 21 (0.09) |  |  |  |  |  |
| **IL3 rs40401** | **CC** | 121 (0.49) | 114 (0.48) |  | **C** | 343 (0.7) | 329 (0.69) | 0.841 |
|  | **CT** | 101 (0.42) | 101 (0.43) | 0.952 | **T** | 147 (0.3) | 145 (0.31) |  |
|  | **TT** | 23 (0.09) | 22 (0.09) |  |  |  |  |  |

**REFERENCES FOR METANALYSIS**

**IL1A**

Katila H, Hänninen K, Hurme M. Polymorphisms of the interleukin-1 gene complex in schizophrenia. *Mol Psychiatry.* 1999; 4(2):179-81.

Chowdari KV, Xu K, Zhang F, *et al.* Immune related genetic polymorphisms and schizophrenia among the Chinese. *Hum Immunol.* 2001;62(7):714-24.

Saiz PA, Garcia-Portilla MP, Arango C, *et al.* Interleukin-1 gene complex in schizophrenia: an association study. *Am J Med Genet B Neuropsychiatr Genet.* 2006;141B(6):678-80.

Watanabe Y, Nunokawa A, Kaneko N, *et al.* Lack of association between the interleukin-1 gene complex and schizophrenia in a Japanese population. *Psychiatry Clin Neurosci.*2007;61(4):364-9

**IL6**

Paul-Samojedny M, Kowalczyk M, Suchanek R, *et al.* Functional polymorphism in the interleukin-6 and interleukin-10 genes in patients with paranoid schizophrenia--a case-control study*. J Mol Neurosci.* 2010;42(1):112-9.

Zakharyan R, Petrek M, Arakelyan A, *et al.* Interleukin-6 promoter polymorphism and plasma levels in patients with schizophrenia. *Tissue Antigens*. 2012; 80(2):136-42.

Paul-Samojedny M, Owczarek A, Kowalczyk M, *et al.* Association of interleukin 2 (IL-2), interleukin 6 (IL-6), and TNF-alpha (TNFα) gene polymorphisms with paranoid schizophrenia in a Polish population*. J Neuropsychiatry Clin Neurosci.* 2013;25(1):72-82

Debnath M, Mitra B, Bera NK, *et al.* Lack of association of IL-6 (-174 G>C) and TNF-α (-238 G>A) variants with paranoid schizophrenia in Indian Bengalee population. *Cytokine.* 2013; 61 (2):455-8

**TNFA**

Shirts BH, Bamne M, Kim JJ, *et al.* A comprehensive genetic association and functional study of TNF in schizophrenia risk. *Schizophr Res.* 2006;83(1):7-13.

Duan S, Xu Y, Chen W, *et al.* No association between the promoter variants of tumor necrosis factor alpha (TNF-alpha) and schizophrenia in Chinese Han population. *Neurosci Lett.* 2004; 366(2):139-43.

Pae CU, Serretti A, Artioli P, *et al.* Interaction analysis between 5-HTTLPR and TNFA -238/-308 polymorphisms in schizophrenia. *J Neural Transm (Vienna).* 2006;113(7):887-97

Watanabe Y, Muratake T, Kaneko N, *et al.* No association between the tumor necrosis factor-alpha gene promoter polymorphisms and schizophrenia in a Japanese population. *Psychiatry Res.* 2007;153(1):1-6.

Betcheva ET, Mushiroda T, Takahashi A, *et al.* Case-control association study of 59 candidate genes reveals the DRD2 SNP rs6277 (C957T) as the only susceptibility factor for schizophrenia in the Bulgarian population. *J Hum Genet.* 2009;54(2):98-107.

Boin F, Zanardini R, Pioli R, *et al.* Association between -G308A tumor necrosis factor alpha gene polymorphism and schizophrenia. *Mol Psychiatry*. 2001;6(1):79-82.

Czerski PM, Rybakowski F, Kapelski P, *et al.*Association of tumor necrosis factor -308G/A promoter polymorphism with schizophrenia and bipolar affective disorder in a Polish population. *Neuropsychobiology*. 2008;57(1-2):88-94.

Dai JP, Zhao ZH, Cai,GH *et al*. "Association analysis of tumor necrosis factor alpha promoter polymorphisms with schizophrenia."*Chinese J Behav Medic Science* 17 (2008): 625-27.

Duan S, Xu Y, Chen W, *et al.* No association between the promoter variants of tumor necrosis factor alpha (TNF-alpha) and schizophrenia in Chinese Han population. *Neurosci Lett.* 2004; 366 (2):139-43.

Hänninen K, Katila H, Rontu R, *et al.* Tumor necrosis factor-alpha --G308A polymorphism in schizophrenia in a Finnish population. *Neurosci Lett.* 2005; 385(1):76-81.

Hashimoto R, Yoshida M, Ozaki N, *et al.* Association analysis of the -308G>A promoter polymorphism of the tumor necrosis factor alpha (TNF-alpha) gene in Japanese patients with schizophrenia. *J Neural Transm (Vienna).* 2004;111(2):217-21.

Kampman O, Anttila S, Illi A, *et al.* Interaction of tumor necrosis alpha - G308A and epidermal growth factor gene polymorphisms in early-onset schizophrenia. *Eur Arch Psychiatry Clin Neurosci.* 2005;255(4):279-83

Meira-Lima IV, Pereira AC, Mota GF,*et al.* Analysis of a polymorphism in the promoter region of the tumor necrosis factor alpha gene in schizophrenia and bipolar disorder: further support for an association with schizophrenia. *Mol Psychiatry.* 2003;8(8):718-20.

Naz M, Riaz M, Saleem M. Potential role of Neuregulin 1 and TNF-alpha (-308) polymorphism in schizophrenia patients visiting hospitals in Lahore, Pakistan. *Mol Biol Rep.* 2011;38(7):4709-14

Pae CU, Chae JH, Bahk WM, *et al.* Tumor necrosis factor-alpha gene polymorphism at position -308 and schizophrenia in the Korean population. *Psychiatry Clin Neurosci*. 2003;57(4):399-403

Riedel M, Krönig H, Schwarz MJ, *et al.* No association between the G308A polymorphism of the tumor necrosis factor-alpha gene and schizophrenia. *Eur Arch Psychiatry Clin Neurosci.* 2002; 252(5):232-4

Sacchetti E, Bocchio-Chiavetto L, Valsecchi P, *et al*. -G308A tumor necrosis factor alpha functional polymorphism and schizophrenia risk: meta-analysis plus association study. *Brain Behav Immun.* 2007;21(4):450-7.

Tan EC, Chong SA, Tan CH, *et al*. Tumor necrosis factor-alpha gene promoter polymorphisms in chronic schizophrenia. *Biol Psychiatry*. 2003;54(11):1205-11

Tsai SJ, Hong CJ, Yu YW, *et al*. No association of tumor necrosis factor alpha gene polymorphisms with schizophrenia or response to clozapine. *Schizophr Res*. 2003;65(1):27-32.

Zai G, Müller DJ, Volavka J, *et al*. Family and case-control association study of the tumor necrosis factor-alpha (TNF-alpha) gene with schizophrenia and response to antipsychotic medication. *Psychopharmacology (Berl).* 2006;188(2):171-82.
